# Fatal Aortoesophageal Fistula Complicating Placement of a 20-mm Lumen-Apposing Metal Stent for Refractory Esophagojejunal Anastomotic Stricture

**DOI:** 10.14309/crj.0000000000000548

**Published:** 2021-03-19

**Authors:** Rupal H. Patel, Brendan T. Everett, Dmitriy Akselrod, Joseph D. Frasca, Stuart R. Gordon

**Affiliations:** 1 Section of Gastroenterology and Hepatology, Dartmouth-Hitchcock Medical Center, Lebanon, NH; 2 Division of Gastroenterology, University of Vermont Medical Center, Burlington, VT; 3 Department of Radiology, University of Vermont Medical Center, Burlington, VT

## Abstract

We report the case of a patient with a benign refractory esophagojejunal anastomotic stricture for which a 20-mm lumen-apposing metal stent was placed, resulting in a fatal aortoenteric fistula. We report this case to alert others to this potential complication of LAMS placement for esophageal strictures and recommend caution when using the 20-mm LAMS in similar settings.

## INTRODUCTION

Endoscopists perform stenting of the esophagus for a wide array of diseases, including focal refractory benign strictures. Until the arrival of lumen-apposing metal stents (LAMSs), we have only had the option of using the traditional longer esophageal stents for this purpose. The use of LAMS for focal refractory benign esophageal strictures is off-label but has been described and is viewed favorably.^1–4^ Because of their safety profile and favorable results, the use of LAMS for refractory benign strictures is increasing. Complications from traditional esophageal stent placement can occur, one of the most severe being an aortoesophageal fistula. This has been reviewed by Zhan and Xu in a recent article, with a total of 14 cases of aortoesophageal fistula reported in the literature after traditional esophageal stent placement.^5^ We report the case of an aortoesophageal fistula after LAMS placement for a refractory benign stricture, which is the first reported case in the literature.

## CASE REPORT

A 66-year-old man with a history of adenocarcinoma of the gastroesophageal junction status-post total gastrectomy with esophagojejunal anastomosis complicated by postoperative anastomotic leak followed by the development of an anastomotic stricture refractory to previous temporary traditional 23-mm esophageal stent placement and multiple endoscopic balloon dilations underwent placement of a 20-mm LAMS (LAMS; Boston Scientific, Marlborough, MA) across the esophagojejunal stricture with plans to leave it in place for 12 weeks. The stricture was 3 mm in length, with an inner diameter of 9 mm, and was located at 38 cm from the incisors. We decided to place a 20-mm LAMS instead of a 15-mm LAMS because the patient previously had tolerated a 23-mm diameter fully covered esophageal stent and had undergone repeated balloon dilations to as high as 18 mm.

**Figure 1.**
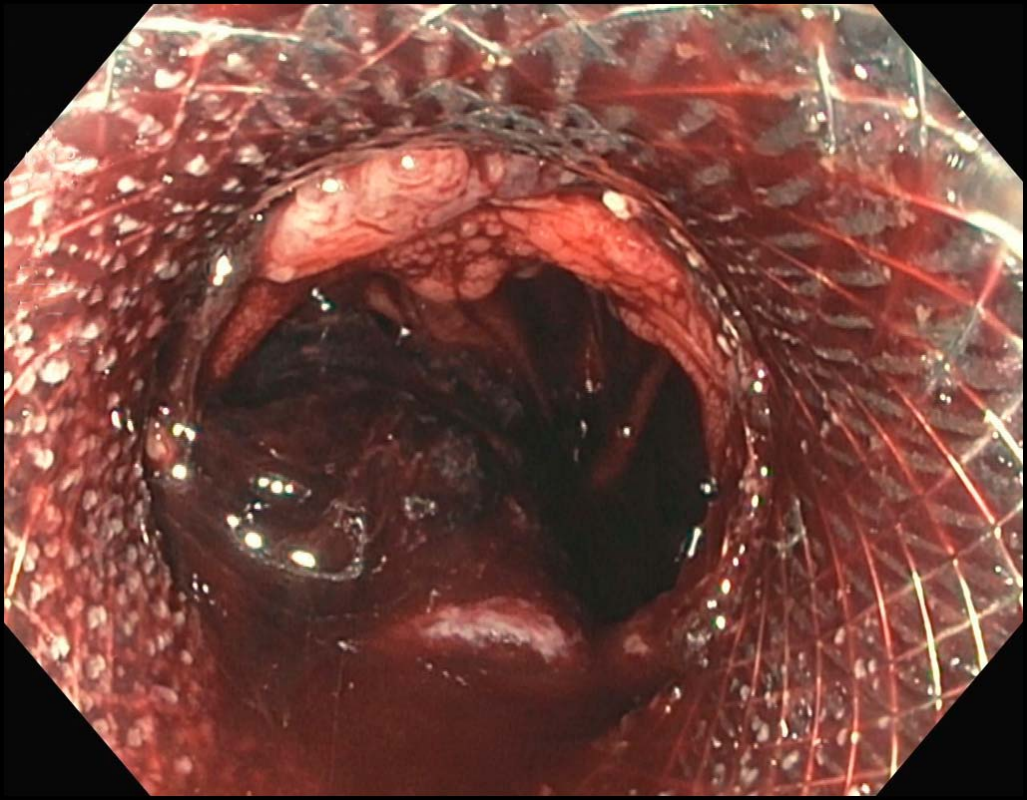
Image from upper endoscopy which revealed the previously placed lumen-apposing metal stent with a rapid arterial hemorrhage from the distal edge of the stent.

**Figure 2.**
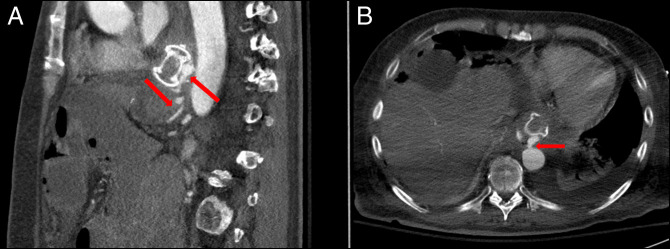
(A) Sagittal view of the computerized tomography angiogram which illustrates an aortoenteric fistula adjacent to the lumen-apposing metal stent (LAMS). There is communication directly from the supraceliac aorta into the lumen of the esophagus/jejunum at the region of the anastomosis and LAMS, with extravasation and pooling of contrast around the LAMS (right arrow) and in the jejunum (left arrow). (B) Axial view of the computerized tomography angiogram again illustrating the aortoenteric fistula (arrow) and the adjacent LAMS.

**Figure 3.**
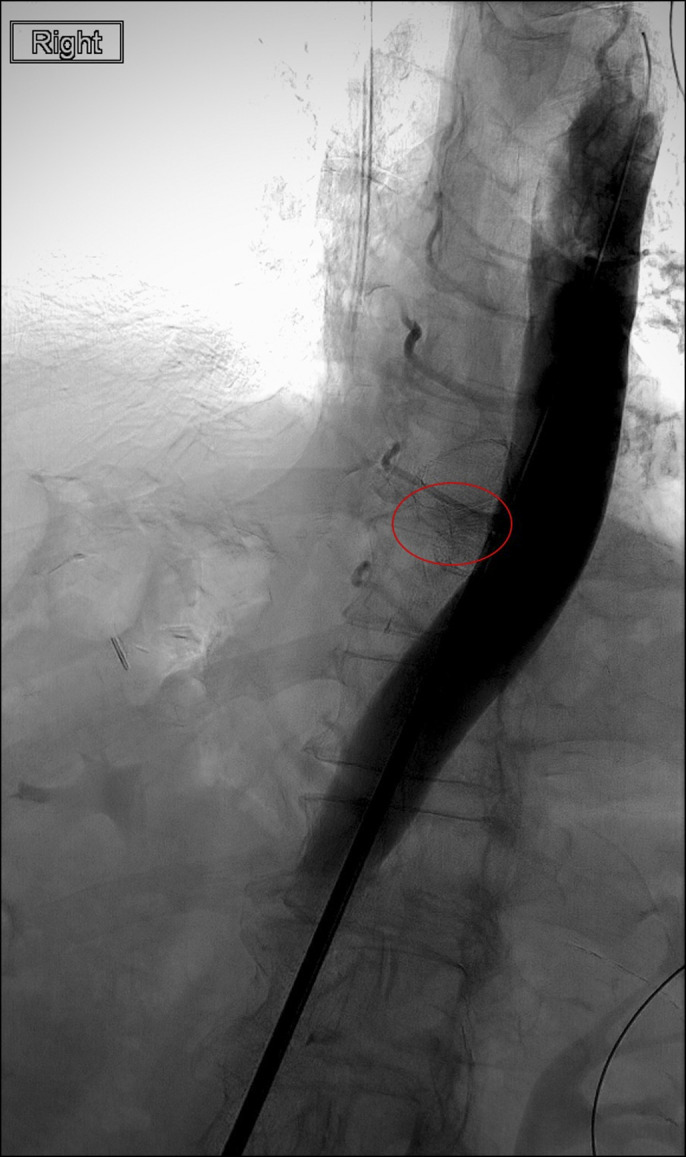
An arteriogram was performed in the OR by surgery colleagues which confirmed an aortoenteric fistula. A blush of contrast originating from the aorta can be seen within the red circle. The lumen-apposing metal stent is also visible in close proximity to the extravasating contrast.

**Figure 4.**
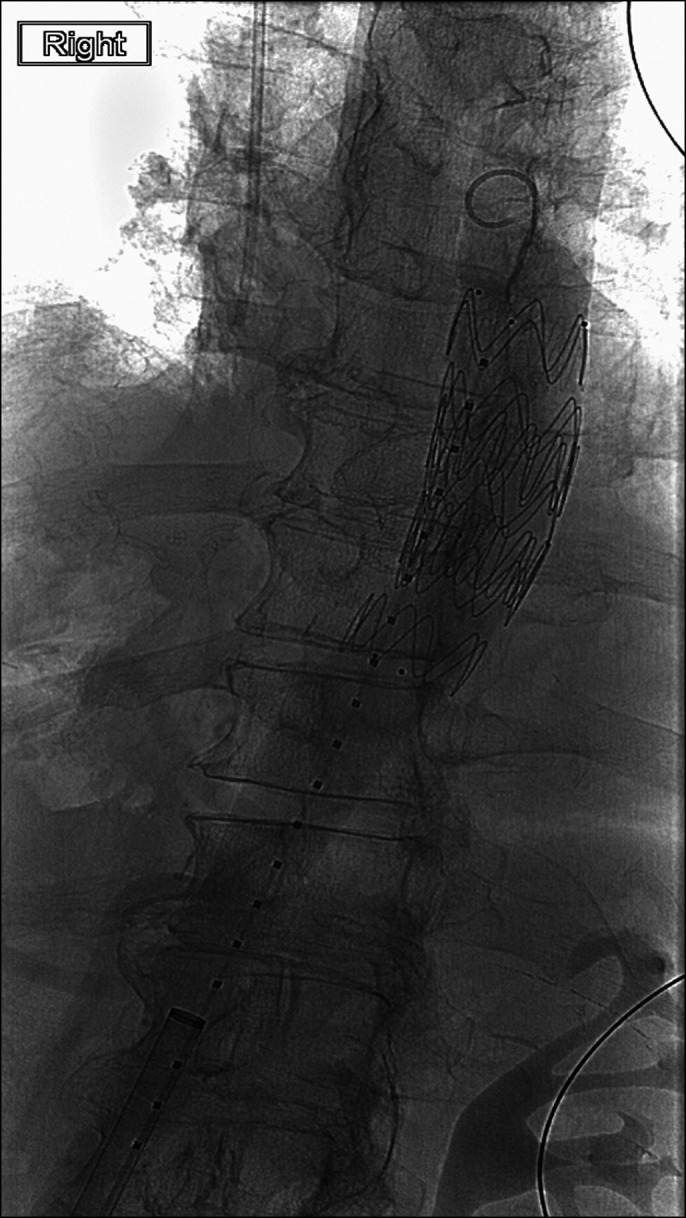
This image shows the aortic stent which was placed to treat the aortoenteric fistula.

Immediately after placement of the LAMS, his dysphagia improved and he was able to tolerate soft foods. Six weeks after stent placement, he presented with hematemesis and hemodynamic instability. An upper endoscopy revealed rapid arterial hemorrhage from the distal edge of the LAMS (Figure 1). Computerized tomography angiogram revealed communication directly from the supraceliac aorta into the lumen of the esophagus/jejunum at the region of the anastomosis and endoluminal stent with active extravasation and pooling of contrast around the stent and jejunum consistent with an aortoenteric fistula (Figure 2). An arteriogram revealed a leak of contrast from the aorta around the LAMS, adjacent to an intercostal artery (Figure 3). The aortoenteric fistula was treated with an endovascular stent graft (Figure 4). The patient subsequently developed septic shock from *Escherichia coli* bacteremia and expired the day after presentation.

## DISCUSSION

In the United States, LAMSs are currently available in 3 sizes (10 mm, 15 mm, and 20 mm) and are increasingly being used to treat benign gastrointestinal strictures refractory to conventional therapy.^1–4^ Although aortoesophageal fistula occurrence has been described after traditional esophageal stent placement, this is the first reported case in the setting of LAMS.^5^ The 20-mm LAMS has a flange diameter of 29 mm which is the largest flange diameter of any esophageal stent. The radial force exerted by this flange width may have resulted in localized ischemia and necrosis followed by development of an aortoesophageal fistula. We report this case to alert others to this potential complication of LAMS placement for esophageal strictures and recommend caution when using the 20-mm LAMS in similar settings. Previous studies have shown symptomatic and endoscopic improvement of benign esophageal strictures using a 15-mm LAMS.^1–4^ Therefore, it might be best to avoid using the 20-mm LAMS for benign refractory esophageal strictures and instead use the smaller caliber 15-mm LAMS.

## DISCLOSURES

Author contributions: All authors contributed equally to this manuscript. RH Patel is the article guarantor.

Financial disclosure: SR Gordon is a consultant for Boston Scientific.

Informed consent was obtained from the patient's next of kin for this case report.
